# Adenosine receptor A2b confers ovarian cancer survival and PARP inhibitor resistance through IL‐6‐STAT3 signalling

**DOI:** 10.1111/jcmm.17802

**Published:** 2023-06-06

**Authors:** Liqing Chi, Lin Huan, Chunyan Zhang, Hanming Wang, Jian Lu

**Affiliations:** ^1^ Institute of Molecular Medicine, College of Future Technology Peking University Beijing China; ^2^ Department of General Surgery, Ruijin Hospital Shanghai Jiao Tong University School of Medicine Shanghai China; ^3^ Fudan University Shanghai Cancer Center and Institutes of Biomedical Sciences, Shanghai Medical College Fudan University Shanghai China

**Keywords:** adenosine, *Adora2b*, IL‐6‐STAT3 signalling, ovarian cancer, PARPi resistance

## Abstract

Ovarian cancer is the deadliest gynecologic cancer worldwide, and the therapeutic options are limited. PARP inhibitor (PARPi) represents an effective therapeutic strategy and has been approved for maintenance therapy. However, the intrinsic or acquired resistance to PARPi becomes a big challenge. To investigate the mechanisms for PARPi resistance, we analysed public databases and established Olaparib‐resistant ovarian cancer cells for exploration. Our results showed that the inflammatory pathway and adenosine receptor A2b (*Adora2b*/A_2B_) expression were significantly increased in Olaparib‐resistant cells. A_2B_ was highly expressed in recurrent ovarian tumours and negatively correlated with the clinical outcomes in cancer patients. Olaparib treatment enhanced A_2B_ expression through NF‐κB activation. The elevated A_2B_ contributed to Olaparib resistance by sensing adenosine signal and promoting tumour cell survival, growth and migration via IL‐6‐STAT3 signalling. Therefore, inhibition of A_2B_‐IL‐6‐STAT3 axis could overcome Olaparib resistance and synergize with Olaparib to reduce cancer cell growth and lead to cell death. Our findings reveal a critical role of A_2B_ signalling in mediating PARPi resistance independent of DNA damage repair, providing insights into developing novel therapies in ovarian cancers.

## 1 INTRODUCTION

Ovarian cancer is the third most common gynecologic cancer worldwide but causes the highest death rate among these cancers.^1^ According to the histological classification, the most frequent type of ovarian cancer is the high‐grade serous ovarian carcinoma (HGSOC) which accounts for approximately 70% of ovarian cancer cases and causes the majority (90%) of cancer‐associated deaths.^2^ Nearly 50% of HGSOCs exhibit defects in homologous recombination (HR), especially *BRCA* *1/2* mutations.^3^ Unfortunately, the treatment of HGSOC remains a big challenge. Platinum‐based chemotherapy (with or without bevacizumab) shows limited response in advanced ovarian cancers.^4^ Currently, poly (ADP‐ribose) polymerase (PARP) inhibitors (PARPis) exhibit good therapeutic outcomes and have been approved for maintenance therapy for ovarian cancer patients.^5^ Olaparib is the first Food and Drug Administration (FDA)‐approved PARPi for ovarian cancers.^6^ Currently, PARPis have been demonstrated beneficial for all ovarian cancer patients regardless of *BRCA* mutation or HR status.^7^, ^8^, ^9^, ^10^

PARPs are involved in DNA damage repair in which they sense the single‐strand breaks and generate large amounts of poly (ADP‐ribose) polymers (PARs) for recruitment of other DNA damage repair participants. Inhibition of PARPs results in accumulation of unrepaired single‐strand breaks and subsequent double‐strand breaks which initiate the HR repair pathways. However, in cells with defects in HR repair pathway, such as *BRCA* mutations, PARPi causes accumulation of double‐strand breaks, leading to cell death. Hence, PARPi has been implemented in *BRCA* mutant tumour cells as an anti‐tumour strategy known as synthetic lethality.^11^ In addition, PARPs participate in regulating inflammatory signalling pathway. PARPs upregulate the expression of several inflammatory molecules such as cytokines, chemokines and transcription factors (mainly NF‐κB) to promote inflammatory responses. Thus, PARPi also represents an effective therapeutic strategy to attenuate inflammation in both inflammatory diseases and cancers.^12^, ^13^ However, long‐term PARPi triggers tumour‐intrinsic DNA damage, leading to the accumulation of DNA errors and activation of DNA‐sensing type I interferon pathways.^14^, ^15^

Despite the potent efficacy in the clinic, almost all ovarian cancer patients received PARPi treatment relapse due to intrinsic or acquired resistance. To date, multiple mechanisms have been identified to contribute to PARPi resistance.^16^, ^17^ Given its primary role in DNA damage repair, much attention has been focused on alternative DNA damage repair pathways. For instance, cancer cells can restore HR repair capacity by decreasing 53BP1 or increasing RAD51 activity to bypass the lethal effects caused by DNA damage accumulation.^18^, ^19^ Additionally, increased drug efflux and re‐establishment of replication fork stability also participate in PARPi resistance.^20^ Nevertheless, it is noteworthy that approximately 50% of HGSOC patients with unaffected HR function in tumour cells can benefit from PARPi treatment,^9^, ^10^ suggesting that more mechanisms beyond DNA damage repair may also play a role in PARPi resistance.

Adenosine (Ado) is accumulated in tumour microenvironment and aggravates tumorigenesis through either promoting cancer cell growth and invasion or inhibiting immune responses.^21^ Four receptors have been characterized for adenosine, namely Adora (A) _1_, A_2A_, A_2B_ and A_3_. With high affinities for adenosine, the receptors A_1_, A_2A_ and A_3_ can sense a low concentration of adenosine at the steady state under physiological conditions in most tissues.^22^ However, A_2B_ has a much lower affinity for adenosine, thus can only respond to adenosine upon pathological stresses, such as hypoxia,^23^, ^24^ ischemia,^25^, ^26^, ^27^, ^28^, ^29^ inflammation^30^ and tumorigenesis^31^, ^32^, ^33^ when both adenosine and A_2B_ expression are increased. Furthermore, four adenosine receptors display antagonistic functions in regulating downstream adenylate cyclase activity and intracellular cAMP levels. While A_2A_ and A_2B_ act with G_s_ to activate adenylate cyclase and increase cAMP levels, A_1_ and A_3_ act with G_i/o_ to inhibit adenylate cyclase activity and decrease cAMP levels.^34^

In this study, we revealed a critical role of A_2B_ signalling in mediating Olaparib resistance in ovarian cancer cells. We found that inflammatory responses and A_2B_ expression were significantly increased in Olaparib‐resistant cancer cells. Olaparib treatment induced A_2B_ upregulation through NF‐κB signalling. The elevated A_2B_ further promoted tumour cell growth and migration by activating IL‐6‐STAT3 signalling, which contributed to Olaparib resistance. Combined inhibition of A_2B_‐IL‐6‐STAT3 signalling and Olaparib could overcome PARPi resistance and exert superior anti‐tumour effects.

## 2 MATERIALS AND METHODS

### 2.1 Cell lines and cell culture

OVCAR3 (ATCC, #HTB‐161), SKOV3 (ATCC, #HTB‐77) and Hey (Pricella, #CL‐0671) cells were cultured in RPMI‐1640 (Gibco, #A1049101) medium. IGROV1 (Sigma, #SCC203), KGN (Pricella, #CL‐0603) and HEK 293 T (ATCC, #CRL‐3216) cells were cultured in DMEM (Gibco, #A11965‐092) medium. PA1 (Cobioer, #CBP60800) cells were cultured in MEM (Gibco, #11095080) medium. All mediums were supplemented with 10% fetal bovine serum (Gibco, #10099‐141C) and 1% penicillin/streptomycin (Gibco, #15140–122). A stable Olaparib‐resistant OVCAR3 (OVCAR3‐R) cell strain was generated by culturing OVCAR3 cells in the continued presence of 12.5 μM Olaparib (Selleck, #S1060) for more than 12 months. All cells were cultured at 37°C in a humidified atmosphere with 5% CO_2_.

### 2.2 RNA‐seq data processing

RNA‐sequencing (RNA‐seq) data of Olaparib‐resistant and parental cells were obtained from GSE153867 and GSE117765. The raw data were aligned to reference using STAR, assembled and quantified by StringTie. Transcripts per million (TPM) was used to evaluate gene expression across samples. Wilcoxon test was used to generate *p*‐values and evaluate the differentially expressed genes.

Gene set enrichment analysis (GSEA) was conducted using the pre‐ranked method. After gene expression being quantified by TPM, log2 scaled fold change of all genes of transcriptome was calculated. C2 (curated genesets), C5 (GO genesets), C6 (oncogenic signatures) and hallmark genesets from MSigDB (Molecular Signatures Database) were analysed. Graphic representations of results were generated using the clusterProfiler package in R (https://www.r‐project.org/).

RNA‐seq data of *Adora2b* (A_2B_) in primary and recurrent tumours were collected from the Santa Cruz Xena online platform (https://xenabrowser.net) using combined datasets of The Cancer Genome Atlas (TCGA), Therapeutically Applicable Research to Generate Effective Treatments (TARGET) and Genotype–Tissue expression (GTEx). Overall survival analysis was performed on Gene Expression Profiling Interactive Analysis (GEPIA) online platform (http://gepia.cancer‐pku.cn/index.html), the *Adora2b* (A_2B_) expression threshold of 50% (median value) was set to split the high‐ and low‐ expression groups, the hazard ratio was calculated based on Cox PH model, and 95% confidence intervals were added as dotted lines.

### 2.3 Plasmid construction, lentivirus production and A_2B_ ‐overexpressed/knockdown cell generation

Complementary DNA (cDNA) of *Adora2b* (NM_000676.4) was cloned into pLVX‐IRES‐Puro plasmid (Addgene) for generation of A_2B_‐overexpressed cells. shRNAs (shAdora2b‐1: sense: 5′‐CCGGGAGCTCCATCTTCAGCCTTCTTCAAGAGAAGGCTGAAGATGGAGCTCTTTTTT‐3′, antisense: 5′‐AATTAAAAAAGAGCTCCATCTTCAGCCTTCTCTTGAAGAAGGCTGAAGATGGAGCTC‐3′ and shAdora2b‐2: sense: 5′‐CCGGGCTGGTGATCTACATTAAGATTCAAGATCTTAATGTAGATCACCAGCTTTTTT‐3′, antisense: 5′‐AATTAAAAAAGCTGGTGATCTACATTAAGATCTTGAATCTTAATGTAGATCACCAGC‐3′) were synthesized and cloned into pLKO.1 puro plasmid (Addgene) for generation of A_2B_‐knockdown cells. All these plasmids including their empty vector controls were separately transfected (together with packaging plasmids pMD2.G and psPAX2, Addgene) into HEK 293 T cells to produce lentivirus. Cell supernatants containing viruses were collected at 48 and 72 h after transfection. For generation of A_2B_‐overexpressed and knockdown cells, OVCAR3 cells were co‐cultivated with supernatants containing viruses and 8 μg/mL polybrene. After 48 h, cells transfected with viruses were selected under 1 μg/mL puromycin.

### 2.4 RNA isolation and RT‐qPCR

Total RNA was isolated using Quick‐RNA™ Microprep Kit (Zymo Research, #R1051) according to the manufacturer's instructions. RNA (0.5–1 μg) was reverse‐transcribed to generate cDNA using PrimeScript™ II 1st Strand cDNA Synthesis Kit (Takara, #6210A). cDNA was subjected to real‐time quantitative polymerase chain reaction (RT‐qPCR) with SYBR™ Green PCR Master Mix (Applied Biosystems, #4309155) on a QuantStudio 5 (Applied Biosystems). Relative expression of mRNAs was normalized to expression of internal control β‐Actin using the 2^(−ΔΔCT)^ method. Primers for RT‐qPCR used in this study were as follows: A_2B_‐*Adora2b* (sense: 5′‐GAGCTCCATCTTCAGCCTTCT‐3′, antisense: 5′‐CGTGACCAAACTTTTATACCTGAGC‐3′); IL‐6‐*Il6* (sense: 5′‐ CAATATTAGAGTCTCAACCCCCA‐3′, antisense: 5′‐ CCGTCGAGGATGTACCGAAT‐3′); β‐Actin‐*Actb* (sense: 5′‐ TTGTTACAGGAAGTCCCTTGCC‐3′, antisense: 5′‐ ATGCTATCACCTCCCCTGTGTG‐3′).

### 2.5 Immunoblotting

Cells were lysed in RIPA lysis buffer (Thermo Scientific, #89900) with protease and phosphatase inhibitor cocktail (Thermo Scientific, #78442). Nuclear proteins were extracted following the protocol of the nuclear protein extraction kit (Beyotime, #P0027). Proteins were separated on SDS‐polyacrylamide gels and electro‐transferred onto polyvinylidene fluoride (PVDF) membranes (Millipore) following standard protocols. After blocking with 5% skimmed milk, membranes were incubated with primary antibodies anti‐A_2B_ (Abcam, #ab229671), anti‐p‐STAT3 (Cell Signaling Technology, #9145), anti‐STAT3 (Cell Signaling Technology, #12640), anti‐p‐NF‐κB p65 (Cell Signaling Technology, #3033), anti‐NF‐κB p65 (Cell Signaling Technology, #8242), anti‐Histone H3 (Proteintech, #17168‐1‐AP) or anti‐β‐Actin (Cell Signaling Technology, #3700) at 4°C overnight, followed by incubation with HRP‐conjugated secondary antibodies. An ECL Plus Western Blotting Substrate (Thermo Scientific, #32134) was used for visualization in an ChemiDoc MP imaging system (Bio‐Rad). ImageJ software was used for quantifications of protein expression which was normalized to internal control β‐Actin.

### 2.6 Cell growth assays

In colony formation assay, cancer cells were seeded into 6‐well plates with 2000 cells/well, and fresh culture mediums were changed every 2 days. OVCAR3, OVCAR3‐R and SKOV3 cells were cultured for 12 days, and Hey cells were cultured for 8 days before quantification. Colony images were taken after fixation with 10% methanol and staining with crystal violet (Beyotime, #C0121). The stained area (%) covered by colonies was quantified using ImageJ software. In cell growth assays, a real‐time cell analyser (RTCA), the xCELLigence RTCA MP instrument (Agilent Technologies), was applied to monitor cell number by impedance changes in 96‐well E‐plates (Agilent Technologies, #300600910). 50 μL culture medium was added into each well of 96‐well E‐plates to obtain equilibrium followed by plating cells into E‐plates with 4000 cells/well in 50 μL culture medium. E‐plate was placed in a RTCA‐multiplate device at 37°C with 5% CO_2_, and impedance changes were recorded to monitor cellular growth. Cell indexes were recorded automatically every 2 h, and normalized to that at 24 h after cells were seeded into E‐plates.

### 2.7 Cell viability assay

Cell viabilities were also detected using a real‐time cell analyser (RTCA) as described above. For IC50 calculation, increasing doses of Olaparib (Selleck, #S1060) in 100 μL culture medium was added into cells seeded in an E‐plate for 24 h. After Olaparib treatment for indicated time, cell indexes at the endpoint were used for curve graphing and IC50 calculation. For combined PARPi and STAT3i treatment, 100 μM Olaparib (Selleck, #S1060) and/or 40 μM C188‐9 (Selleck, #S8605) in 100 μL culture medium was added into 100 μL cultured cells after seeded in an E‐plate for 24 h. After treatment for 5 days, cell indexes were normalized to that at 24 h after cells were seeded into E‐plates and were used to determine cell viability.

### 2.8 Cell migration assay

Cell migration was measured by the wound closure method. Briefly, a total of 5 × 10^4^ cells per cm^2^ were seeded on 6‐well plates. An artificial wound was scratched into the confluent cell monolayer. The microphotographs were captured before (0 h) and after culture (48 h). The closing area of the scratch in microphotographs was quantified using ImageJ software.

### 2.9 Cytokine detection

The supernatants of cultured cells treated with vehicle or adenosine (Sigma, A9251) were collected. The IL‐6 concentrations in the supernatants were analysed using a HTRF™ human IL‐6 kit (Cisbio Bioassays, #62HIL06PET) according to the manufacturer's instructions. Signals were obtained using a TECAN (Spark) plate reader.

### 2.10 Flow cytometry analysis

Cells treated with vehicle or Olaparib for 24 h were collected and prepared as single cell suspensions. Cells were fixed and permeabilizated using transcription factor staining buffer set (Invitrogen, #00–5523‐00), and stained with Alexa Fluor® 647 conjugated anti‐phosphoNF‐κB p65 (Ser536) antibody (Cell Signalling Technology, #4887S) for 30 min. Data were collected with a FACSCelesta flow cytometer (BD Biosciences) and analysed using FlowJo_V10 software.

### 2.11 Statistical analysis

Data were presented as mean ± standard deviation (SD). The *n* value represents biological replicates. Statistical analyses were performed using GraphPad Prism 8.0 or Microsoft Excel 2019. The two‐tailed unpaired Student's *t*‐test was used for comparisons between two independent groups. Statistical significance of differences between two time‐course curves was determined using two‐way anova. Statistical significance was marked as *p < 0.05, **p < 0.01, ***p < 0.001 or #*p* < 0.05, ##*p* < 0.01.

## 3 RESULTS

### 3.1 Elevated A_2B_ expression in Olaparib‐resistant ovarian cancer cells

**FIGURE 1:**
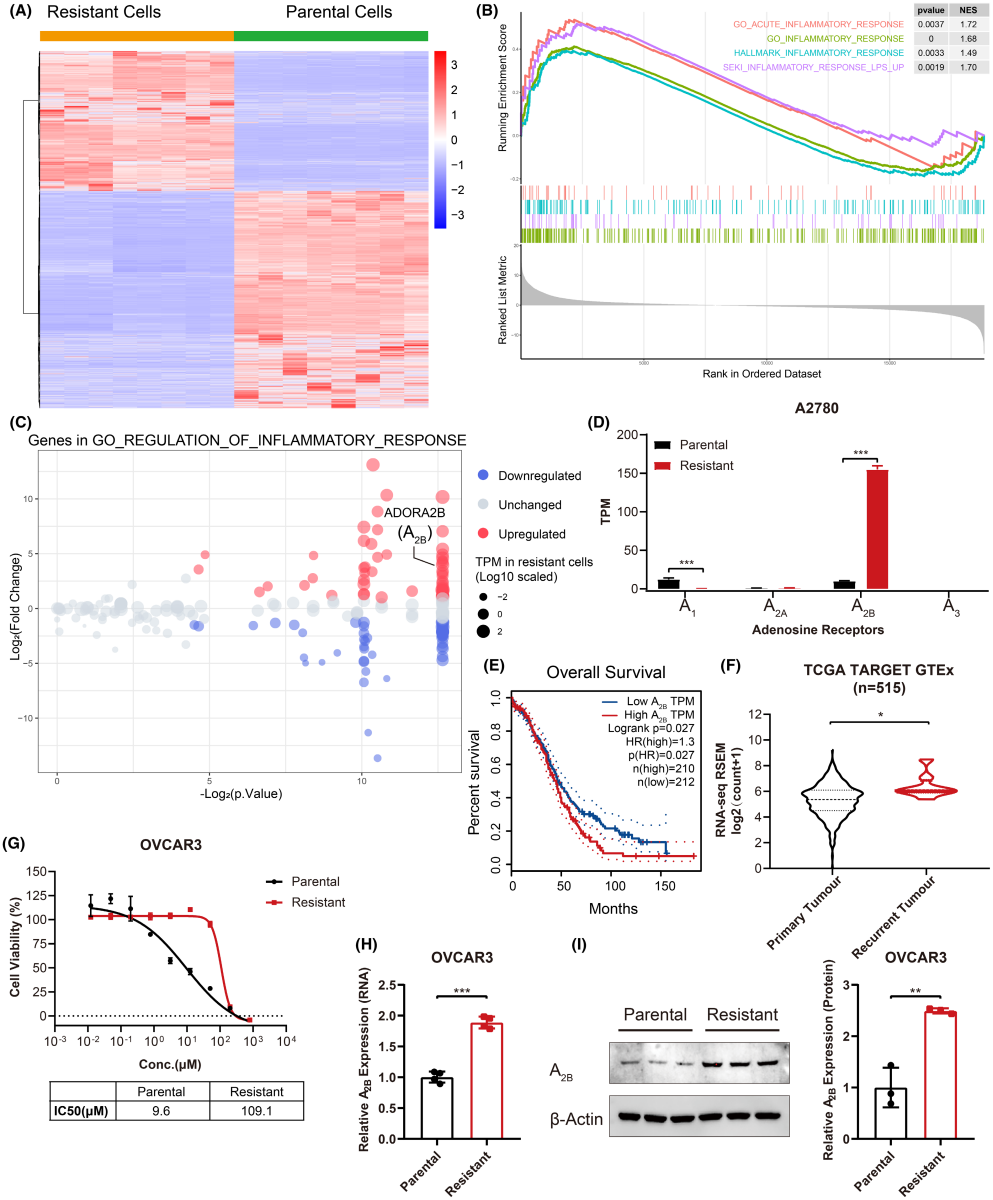
A_2B_ expression is increased in Olaparib‐resistant ovarian cancer cells and correlates with poor prognosis. (A) Heat map illustrating the differentially expressed genes between parental and Olaparib‐resistant A2780 cells (*n* = 8). *p* value was calculated using Wilcoxon test, and genes with |Log_2_ (Fold Change)| > 1 and *p* < 0.05 were chosen for heat map. (B) Gene set enrichment analysis (GSEA) of inflammatory response pathway using GES153864 (PEO1 cell) data. (C) Volcano plot depicting changes of genes in Gene Ontology geneset, *p* value was calculated using Wilcoxon test. (D) Transcripts per million (TPM) values of adenosine receptor genes in parental and Olaparib‐resistant A2780 cells were shown. (E) Overall survival analysis of ovarian cancer patients from TCGA database based on A_2B_ expression. (F) RNA‐seq violin plot exhibiting the expression of A_2B_ (*Adora2b*) in primary and recurrent tumours from ovarian cancer patients. The RNA‐seq data was collected from Santa Cruz Xena platform using the combined TCGA, TARGET and GTEX datasets. (G) Cell growth inhibition was determined in parental (OVCAR3) and Olaparib‐resistant (OVCAR3‐R) cells treated with Olaparib (*n* = 3), and dose–response curves were graphed to calculate IC50 values. (H) Real‐time quantitative PCR (RT‐qPCR) analysis of A_2B_ expression in OVCAR3 and OVCAR3‐R cells (*n* = 4). The relative expression was normalized to A_2B_ expression in parental cells. (I) Immunoblot analysis of A_2B_ expression in OVCAR3 and OVCAR3‐R cells (*n* = 3). The immunoblot image was on the left and the quantification of A_2B_ expression was on the right, in which the relative expression was normalized to A_2B_ expression in parental OVCAR3 cells. Data are representative of three independent experiments shown as the mean ± SD. Statistical testing is depicted as two‐tailed unpaired Student's *t*‐test. *p < 0.05, **p < 0.01, ***p < 0.001.

To explore the alternative mechanisms of PARPi resistance, we retrieved the gene expression data from the GEO database and analysed the transcriptomes of two Olaparib‐resistant ovarian cancer cell lines, A2780 (A2780‐R) and PEO1 (PEO1‐R). Compared to parental cells, Olaparib‐resistant cells showed significantly distinct transcriptomic profiles with large amounts of differentially expressed genes (DEGs) in both A2780‐R (Figure 1A) and PEO1‐R (Figure S1A) cells. The gene set enrichment analysis (GSEA) of DEGs revealed that inflammatory response related pathways were markedly enriched in both A2780‐R (Figure 1B) and PEO1‐R (Figure S1B) cells. Among genes in the inflammatory response pathway, the expression of A_2B_, one of the adenosine receptors, was remarkably increased (Figure 1C and Figure S1C). More importantly, only A_2B_, but not other three adenosine receptors (A_1_, A_2A_ and A_3_) was elevated in Olaparib‐resistant cells (Figure 1D and Figure S1D). Interestingly, as a member of G protein coupled receptors (GPCRs), A_2B_ acts with G_s_ protein to activate cAMP signalling.^34^ However, GPCR signalling was decreased in Olaparib‐resistant cells (Figure S1E), suggesting that A_2B_ may not function through classical GPCR‐cAMP pathways.

The elevated A_2B_ in Olaparib‐resistant ovarian cancer cells intrigued us to explore whether A_2B_ acted as a risk factor for ovarian cancer patients. Based on The Cancer Genome Atlas (TCGA) data, the expression of A_2B_ was negatively correlated with the overall survival (OS) in ovarian cancer patients (Figure 1E). Moreover, the A_2B_ expression was much higher in recurrent ovarian tumours than in primary tumours (Figure 1F), indicating a potential role of A_2B_ in mediating drug resistance in recurrent ovarian cancer. To further study the function of A_2B_ in PARPi resistance, we established the Olaparib‐resistant ovarian cancer cell line OVCAR3‐R which showed significantly increased IC50 value compared to parental cells (Figure 1G) even with long‐time exposure (Figure S2). Consistent with previous results, the expression of A_2B_ was significantly increased in OVCAR3‐R cells at both mRNA (Figure 1H) and protein (Figure 1I) levels. These data suggest that A_2B_ is elevated in Olaparib‐resistant ovarian cancer cells and may play critical roles in PARPi resistance.

### 3.2 Olaparib treatment induces A_2B_ expression through NF‐κB activation

**FIGURE 2:**
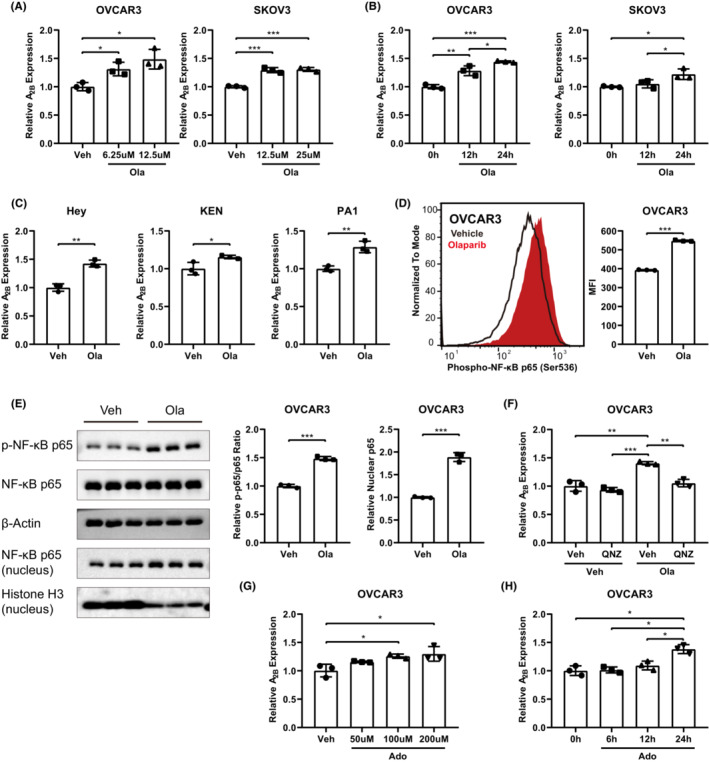
Olaparib treatment elevates A_2B_ expression through activation of NF‐κB. (A) RT‐qPCR analysis of A_2B_ expression in OVCAR3 (left) and SKOV3 (right) cells treated with indicated doses of Olaparib for 24 h (*n* = 3), and the relative expression was normalized to vehicle (Veh) group. (B) RT‐qPCR analysis of A_2B_ expression in OVCAR3 (left) and SKOV3 (right) cells treated with 6.25 μM (OVCAR3) or 12.5 μM (SKOV3) Olaparib for indicated time (*n* = 3), and the relative expression was normalized to A_2B_ expression in cells without Olaparib treatment (0 h). (C) RT‐qPCR analysis of A_2B_ expression in Hey (left), KGN (middle) and PA1 (right) ovarian cancer cells treated with vehicle (Veh) or 12.5 μM Olaparib (Ola) for 24 h (*n* = 3), and the relative expression was normalized to A_2B_ expression in Veh groups. (D) Flow cytometric analysis of phosphorylation of NF‐κB subunit p65 in OVCAR3 cells treated with 12.5 μM Olaparib for 24 h (*n* = 3). Representative overlapping histogram was shown on the left, and statistic graph of mean fluorescence intensity (MFI) was shown on the right. (E) Immunoblot analysis of phosphorylation of NF‐κB subunit p65 in OVCAR3 cells treated with 12.5 μM Olaparib for 30 min (*n* = 3), and immunoblot analysis of nuclear NF‐κB subunit p65 in OVCAR3 cells treated with 12.5 μM Olaparib for 30 min (*n* = 3). The immunoblot image was on the left. The quantification of p‐NF‐κB/NF‐κB ratio was on the middle, in which the relative ratio was normalized to the p‐NF‐κB/NF‐κB ratio in vehicle‐treated OVCAR3 cells. The quantification of nuclear NF‐κB was on the right, in which the relative amount of nuclear NF‐κB was normalized to the amount of Histone H3 in vehicle‐treated OVCAR3 cells. (F) RT‐qPCR analysis of A_2B_ expression in OVCAR3 cells treated with Olaparib (6.25 μM) and/or QNZ (28 nM) for 24 h. The relative expression was normalized to A_2B_ expression in cells treated with vehicle. RT‐qPCR analysis of A_2B_ expression in OVCAR3 cells treated with increasing dose of adenosine (Ado) for 24 h (G), or treated with 100 μM adenosine (Ado) for indicated time (H) (*n* = 3). The relative expression was normalized to A_2B_ expression in cells without adenosine (Ado) treatment. Data are representative of three independent experiments shown as the mean ± SD. Statistical testing is depicted as two‐tailed unpaired Student's *t*–test. *p < 0.05, **p < 0.01, ***p < 0.001.

The increased A_2B_ expression in Olaparib‐resistant ovarian cancer cells can be either innate or acquired drug resistance. To address this issue, we examined the expression of A_2B_ in OVCAR3 and SKOV3 cells with or without Olaparib treatment. The results showed that Olaparib‐induced an increased expression of A_2B_ in dose‐ and time‐dependent manners in both OVCAR3 and SKOV3 cells (Figure 2A,B). Similar results were also observed in other ovarian cell types, including KGN, Hey and PA1 (Figure 2C).

NF‐κB, as a master transcription factor driving pro‐inflammatory responses,^35^ has been reported involved in upregulating A_2B_ expression.^36^, ^37^, ^38^ Given that the inflammatory signalling was significantly increased in resistant cells after long‐term Olaparib treatment (Figure 1B and Figure S1B), we next investigated whether Olaparib could induce A_2B_ expression through activation of NF‐κB. We found that in both OVCAR3 and SKOV3 ovarian cancer cells, Olaparib treatment induced an elevated NF‐κB activation evidenced by both increased phosphorylation of NF‐κB subunit p65 (Figure 2D,E) as well as NF‐κB nuclear translocation (Figure 2E). Additionally, A_2B_ expression was examined after Olaparib treatment with or without NF‐κB inhibition using its specific inhibitor Quinazolinediamine (QNZ). The results showed that while NF‐κB inhibition had no impact on A_2B_ expression in OVCAR3 cells, QNZ treatment significantly inhibited Olaparib‐induced increase of A_2B_ expression (Figure 2F), indicating that Olaparib upregulated the expression of A_2B_ through activation of NF‐κB signalling. As the adenosine receptor, A_2B_ sensed adenosine stimuli and further promoted its expression in both dose‐ and time‐dependent manners (Figure 2G,H), forming a positive feedback loop. Such positive feedback loop might contribute to Olaparib resistance mediated by A_2B_ accumulation in resistant cells.

### 3.3 Upregulated A_2B_ contributes to Olaparib resistance

**FIGURE 3:**
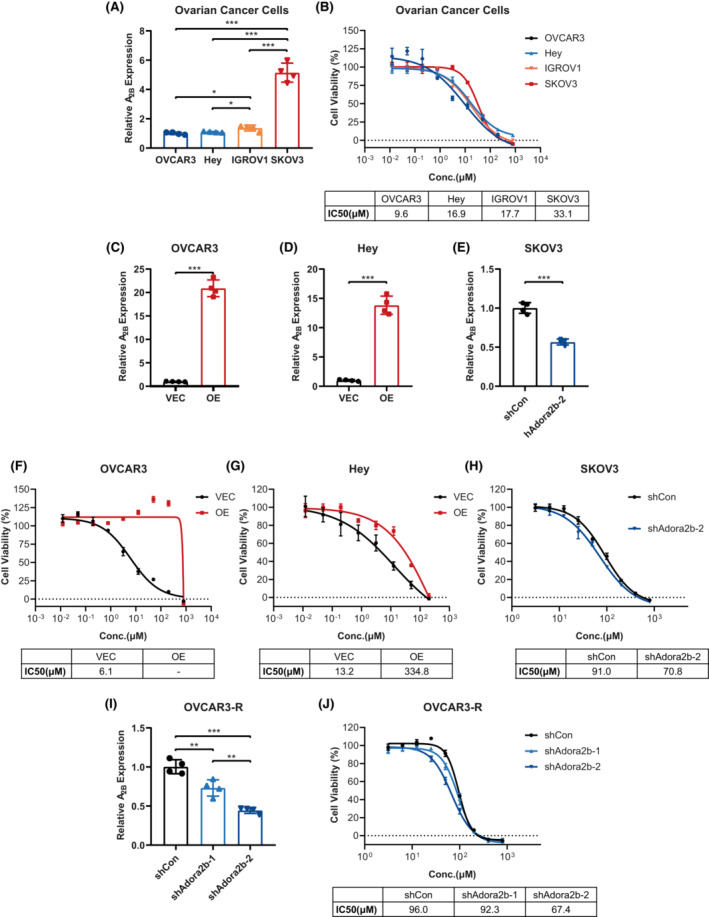
Expression of A_2B_ positively correlates with Olaparib resistance in ovarian cancer cells. (A) RT‐qPCR analysis of A_2B_ expression in OVCAR3, Hey, IGROV1 and SKOV3 ovarian cancer cells (*n* = 4), and the relative expression was normalized to A_2B_ expression in OVCAR3 cells. (B) Cell growth inhibition curves showing ovarian cancer cells OVCAR3, Hey, IGROV1 and SKOV3 treated with different concentrations of Olaparib (*n* = 3), and the dose–response curves were graphed and IC50s were indicated. RT‐qPCR analysis of A_2B_ expression in OVCAR3 cells (C) and Hey cells (D) with (OE) and without (VEC) A_2B_ overexpression (*n* = 4). The relative expression was normalized to VEC groups. (E) RT‐qPCR analysis of A_2B_ expression in SKOV3 cells with (shAdora2b‐2) and without (shCon) A_2B_‐knockdown (*n* = 4), and the relative expression was normalized to shCon group. Cell growth inhibition curves of control (VEC) and A_2B_‐overexpressed (OE) OVCAR3 cells (*n* = 3) (F) and Hey cells (*n* = 3) (G) treated with Olaparib were shown, and the dose–response curves were graphed and IC50s were indicated. (H) Cell growth inhibition curves of SKOV3 cells with (shAdora2b‐2) and without (shCon) A_2B_‐knockdown control (shCon) treated with Olaparib (*n* = 3), and the dose–response curves were graphed and IC50s were indicated. (I) RT‐qPCR analysis of A_2B_ expression in OVCAR3‐R cells with (shAdora2b‐1/2) and without (shCon) A_2B_‐knockdown (*n* = 4), and the relative expression was normalized to control group. (J) Cell growth inhibition curves of OVCAR3‐R cells with (shAdora2b‐1/2) and without (shCon) A_2B_‐knockdown treated with Olaparib (*n* = 3), and the dose–response curves were graphed and IC50s were indicated. Data are representative of three independent experiments shown as the mean ± SD. Statistical testing is depicted as two‐tailed unpaired Student's *t*‐test. *p < 0.05, **p < 0.01, ***p < 0.001.

We next sought to explore whether increased expression of A_2B_ was sufficient to induce Olaparib tolerance. We first compared the expression of A_2B_ in different ovarian cancer cell lines, including OVCAR3, Hey, IGROV1 and SKOV3 (Figure 3A). And we found that as the A_2B_ expression increased, the cells displayed enhanced insensitivity to Olaparib, in which SKOV3 expressing the highest level of A_2B_ had the highest IC50 to Olaparib (Figure 3A,B). These findings suggested a positive correlation between A_2B_ expression and Olaparib resistance. To gain more evidence on this notion, we overexpressed A_2B_ in two low A_2B_‐expressing cell lines OVCAR3 and Hey (Figure 3C,D). As a control, we also knocked down A_2B_ expression in the high A_2B_‐expressing cell line SKOV3 (Figure 3E). As expected, A_2B_ overexpression remarkably elevated the IC50 values in OVCAR3 and Hey cells (Figure 3F,G) whereas A_2B_ knockdown decreased the IC50 in SKOV3 cells (Figure 3H). Moreover, A_2B_ knockdown in OVCAR3‐R cells also significantly decreased the IC50 values in a dose‐dependent manner (Figure 3I,J). Taken together, these results demonstrate that elevated A_2B_ directly contributes to Olaparib resistance.

### 3.4 Upregulated A_2B_ promotes cell growth and migration in ovarian cancer cells

**FIGURE 4:**
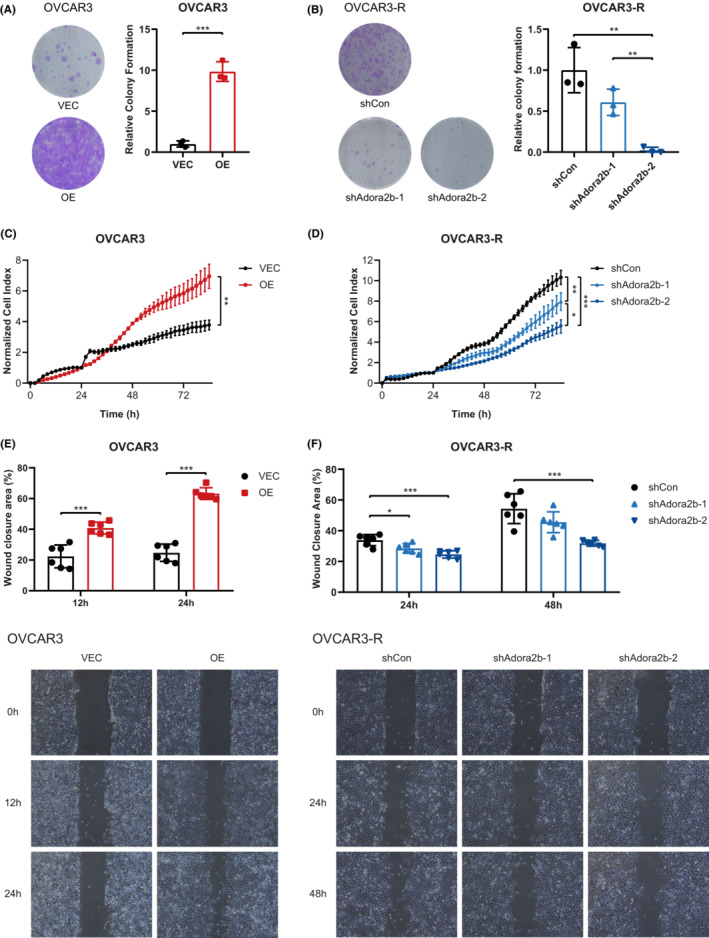
A_2B_ signalling promotes ovarian cancer cell growth and migration. (A) Cell growth of control (VEC) and A_2B_‐overexpressed (OE) OVCAR3 cells was detected by colony formation assay (*n* = 3). The representative images were on the left, and the statistical graph was on the right with normalization to VEC group. (B) Cell growth of OVCAR3‐R cells with (shAdora2b‐1/2) and without (shCon) A_2B_‐knockdown was detected by colony formation assay (*n* = 3). The representative images were on the left, and the statistical graph was on the right with normalization to shCon group. (C) Cell growth of control (VEC) and A_2B_‐overexpressed (OE) OVCAR3 cells was detected using a real‐time cell analyser (*n* = 3). The cell index was normalized to that at 24 h after cells seeded onto plates, and *p* value was calculated using two‐way anova. (D) Cell growth of OVCAR3‐R cells with (shAdora2b‐1/2) and without (shCon) A_2B_‐knockdown was detected using a real‐time cell analyser (*n* = 3). The cell index was normalized to that at 24 h after cells seeded onto plates, and *p* value was calculated using two‐way anova. (E) Cell migration of control (VEC) and A_2B_‐overexpressed (OE) OVCAR3 cells was detected using wound healing assay (n = 6). The statistical graph was shown on the top, and the representative images were shown on the bottom. (F) Cell migration of OVCAR3‐R cells with (shAdora2b‐1/2) and without (shCon) A_2B_‐knockdown was detected using wound healing assay (*n* = 6). Data are representative of three independent experiments shown as the mean ± SD. Statistical testing is depicted as two‐tailed unpaired Student's *t*‐test. *p < 0.05, **p < 0.01, ***p < 0.001.

Previous studies have demonstrated a role of A_2B_ signalling in promoting cell proliferation and migration in some types of tumours.^39^ To determine the function of increased A_2B_ expression in ovarian cancer cells, we examined the cell growth and migration in cells with A_2B_ overexpression and knockdown. The results showed that the colony formation was significantly enhanced after A_2B_ overexpression in OVCAR3 (Figure 4A) and Hey (Figure S3A) cells. On the contrary, A_2B_ knockdown in OVCAR3‐R (Figure 4B) and SKOV3 (Figure S3B) cells exhibited a markedly reduced ability to form colonies. Notably, the expression level of A_2B_ was positively associated with the colony formation capability of ovarian cancer cells as a low A_2B_ expression led to fewer colonies (Figure 4B). Consistently, A_2B_ overexpressed OVCAR3 **(**Figure 4C) and Hey (Figure S3C) cells showed greatly augmented cell growth rates compared with their parental cells, while A_2B_ knockdown in OVCAR3‐R (Figure 4D) and SKOV3 (Figure S3D) cells had reduced cell growth. Besides, OVCAR3‐R cells which had upregulated A_2B_ expression (Figure 1H and Figure 1I) displayed superior cell growth compared to the parental cells (Figure S3E). Furthermore, metastasis is a hallmark of cancer.^40^ We then examined the effect of A_2B_ on ovarian cancer cell migration. The wound healing assay demonstrated that high A_2B_ expression significantly promoted the migratory capability of OVCAR3 cells (Figure 4E), while decreased A_2B_ expression affected the cell migration in OVCAR3‐R cells (Figure 4F). These results elucidate that upregulated A_2B_ expression contributes to Olaparib resistance in ovarian cancer cells likely through promoting cell proliferation and migration.

### 3.5 A_2B_ confers Olaparib resistance in cancer cells via IL‐6‐STAT3 signalling

**FIGURE 5:**
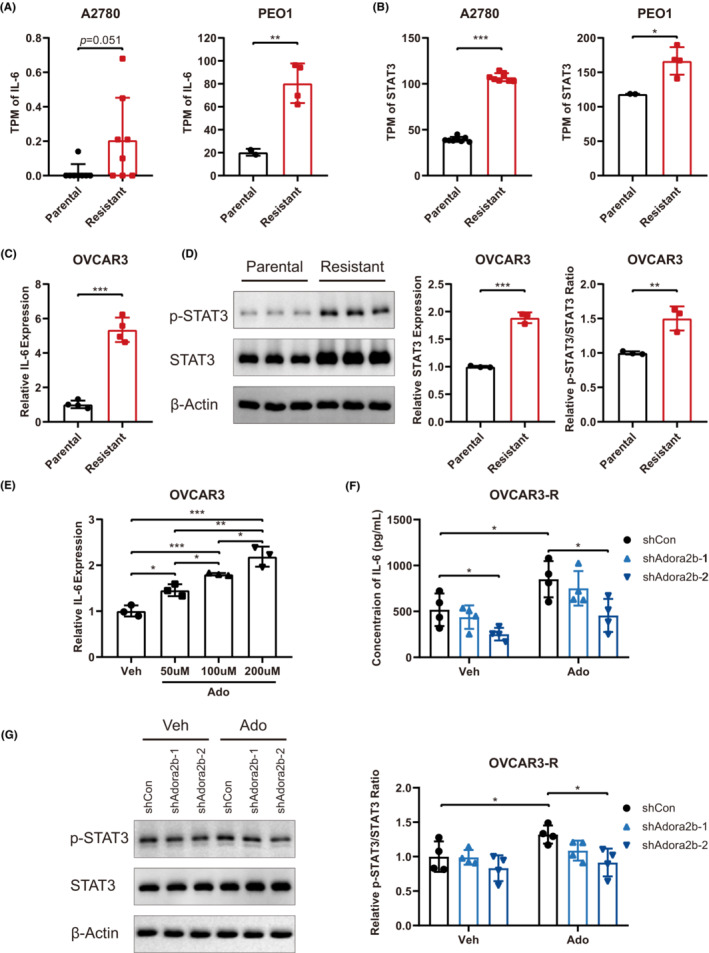
A_2B_ promotes cell growth and Olaparib resistance in ovarian cancer cells through activating IL‐6‐STAT3 pathway. TPM values of *Il6* gene (A) and *Stat3* gene (B) in parental and Olaparib‐resistant A2780 (A and B, left) and PEO1 (A and B, right) cells were shown. (C) RT‐qPCR analysis of IL‐6 expression in parental (OVCAR3) and Olaparib‐resistant (OVCAR3‐R) cells (*n* = 4), and the relative expression was normalized to IL‐6 expression in parental cells. (D) Immunoblot analysis of total and phosphorylated STAT3 expression in OVCAR3 and OVCAR3‐R cells (*n* = 3). The immunoblot image was on the left. The quantification of STAT3 expression was on the middle, in which the relative expression was normalized to STAT3 expression in parental OVCAR3 cells. The quantification of p‐STAT3/STAT3 ratio was on the right, in which the relative ratio was normalized to the p‐STAT3/STAT3 ratio in parental OVCAR3 cells. (E) RT‐qPCR analysis of IL‐6 expression in OVCAR3 cells treated with indicated doses of adenosine (Ado) for 24 h (*n* = 3), and the relative expression was normalized to Veh group. (F) Concentration of IL‐6 in the supernatant of cultured OVCAR3‐R cells with (shAdora2b‐1/2) and without (shCon) A_2B_‐knockdown treated with vehicle (Veh) or 100 μM adenosine (Ado) for 24 h (*n* = 4). (G) Immunoblot analysis of total and phosphorylated STAT3 expression in OVCAR3‐R cells with (shAdora2b‐1/2) and without (shCon) A_2B_‐knockdown treated with vehicle (Veh) or 100 μM adenosine (Ado) for 12 h (*n* = 4). The representative image was on the left. The quantification of p‐STAT3/STAT3 ratio was on the right, in which the relative ratio was normalized to the p‐STAT3/STAT3 ratio in shCon cells treated with vehicle. Data are representative of three independent experiments shown as the mean ± SD. Statistical testing is depicted as two‐tailed unpaired Student's *t*‐test. *p < 0.05, **p < 0.01, ***p < 0.001.

A_2B_ signalling regulates cell survival and proliferation by activating several downstream signalling pathways, including cAMP‐PKA‐p38, PI3K‐ERK1/2, PKC‐JNK and IL‐6‐STAT3.^39^, ^41^ Given the enriched inflammatory pathways in Olaparib‐resistant cancer cells, we focused on the IL‐6‐STAT3 axis which acts as a predominant mediator of inflammation.^42^ Transcriptomic analysis showed that both IL‐6 and STAT3 expression were significantly upregulated in Olaparib‐resistant PEO1‐R and A2780‐R cells (Figure 5A,B). Comparable results were observed in OVCAR3‐R cells (Figure 5C,D). It is well‐appreciated that enhanced STAT3 signalling, including both STAT3 expression and phosphorylation, is critical for cell survival and proliferation.^43^, ^44^, ^45^ IL‐6 stimulated p‐STAT3 activation drives many gene expression, including STAT3 itself.^45^ Thus, both total and phosphorylated/activated STAT3 were elevated in OVCAR3‐R cells at the protein level (Figure 5D). To confirm the involvement of A_2B_ signalling, we treated OVCAR3 cells with A_2B_ ligand adenosine (Ado) and found that activated adenosine signalling could increase the expression of IL‐6 in a dose‐dependent manner (Figure 5E). It is noteworthy that this effect of adenosine on inducing IL‐6 expression has been reported in immune cells but not in cancer cells previously.^41^ Likewise, A_2B_ knockdown in OVCAR3‐R cells reduced IL‐6 production in the supernatant of both vehicle‐ and adenosine‐treated OVCAR3‐R cells (Figure 5F). Furthermore, adenosine treatment remarkably increased STAT3 phosphorylation (p‐STAT3/STAT3 ratio) whereas A_2B_ knockdown reduced the STAT3 phosphorylation (Figure 5G). Taken together, A_2B_ signalling mediates Olaparib resistance via activating IL‐6‐STAT3 signalling in ovarian cancer cells.

### 3.6 A_2B_‐IL‐6‐STAT3 axis inhibition synergizes with Olaparib to repress tumour cell growth

**FIGURE 6:**
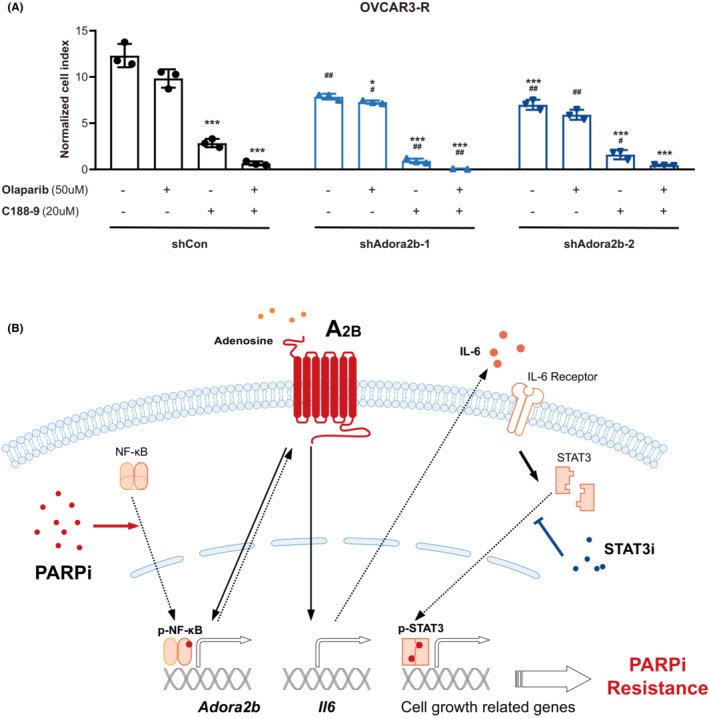
Combination of PARPi with A_2B_‐IL‐6‐STAT3 inhibition exhibits synergetic effects to overcome PARPi resistance. (A) Cell growth of OVCAR3‐R cells with (shAdora2b‐1/2) and without (shCon) A_2B_‐knockdown treated with Olaparib or/and STAT3 inhibitor C188‐9 was detected using a real‐time cell analyser (*n* = 3). The cell index was normalized to that at 24 h after cells seeded onto plates. Data are shown as the mean ± SD. Statistical testing is depicted as two‐tailed unpaired Student's *t*‐test. *p < 0.05 and ***p < 0.001, compared to cells treated with vehicle. #*p* < 0.05 and ##*p* < 0.01, compared to shCon cells. (B) A schematic diagram illustrating that A_2B_ signalling confers PARPi resistance in ovarian cancer cells. Briefly, PARPi (Olaparib) treatment induces upregulation of A_2B_ expression through NF‐κB signalling. The elevated A_2B_ senses adenosine and activates IL‐6‐STAT3 signalling to promote cell growth and migration, contributing to Olaparib resistance. Therefore, inhibition of A_2B_‐IL‐6‐STAT3 axis synergizes with Olaparib to repress tumour cell growth.

Since upregulation of A_2B_ and subsequent activation of IL‐6‐STAT3 signalling endows ovarian cancer cells with Olaparib resistance, we wondered if inhibition of A_2B_‐IL‐6‐STAT3 axis could overcome such resistance and prevent tumour cell growth. To test this hypothesis, we combined the treatment of STAT3 inhibitor in addition to Olaparib in OVCAR3‐R cells with or without A_2B_ knockdown (Figure 6A). C188‐9 is a specific STAT3 inhibitor which has been broadly used in clinical trials. While Olaparib alone, even with a high concentration (50uM), showed limited effects on those resistant cells, C188‐9 could significantly inhibit cell growth (Figure 6A left). Importantly, C188‐9 treatment sensitized the cells to Olaparib, thus cells in combined treatment had the lowest cell growth (Figure 6A left). Moreover, there were statistically significant differences between shCon and shAdora2b groups treated with either C188‐9 alone or together with Olaparib (Figure 6A), indicating that inhibition of A_2B_ expression further promoted the anti‐tumour effects of Olaparib and C188‐9. Collectively, these results demonstrate that inhibition of A_2B_‐IL‐6‐STAT3 signalling overcomes PARPi resistance and synergizes with Olaparib to reduce cancer cell viability.

## 4 DISCUSSION

Olaparib, as the first FDA‐approved PAPR inhibitor, has been widely used in the maintenance therapy of ovarian cancers.^6^ However, a majority of patients have a relapse after primary therapy with PARPi (including Olaparib).^16^, ^20^ Hence, it is of great importance to investigate the mechanisms by which ovarian cancer cells are resistant to Olaparib. In this study, we demonstrate a critical role of A_2B_ in mediating Olaparib resistance (Figure 6B). We reveal that the administration of Olaparib on ovarian cancer cells induces upregulation of A_2B_ expression through NF‐κB signalling. The upregulated A_2B_ senses adenosine signal and activates IL‐6‐STAT3 signalling pathway to promote cell growth and migration, which confers the Olaparib resistance in cancer cells. Thus, inhibition of A_2B_‐IL‐6‐STAT3 axis synergizes with Olaparib to repress tumour cell growth, providing insights into developing novel cancer therapies.

Previously, the mechanisms for PARPi resistance have been largely focused on alternative DNA damage repair.^3^ However, PARPi also exhibits effectiveness in patients with proficient DNA damage repair function.^3^, ^9^, ^10^ Moreover, in HR‐proficient tumour cells, long‐term PARP inhibition induces DNA damage, which initiates DNA‐sensing type I interferon response and inflammation.^14^, ^15^ Therefore, other mechanisms for PARPi resistance in addition to innate DNA damage repair may exist, especially mechanisms related to the newly generated DNA damage and followed inflammatory responses. In our study, the ovarian cancer cell lines (OVCAR3, Hey, SKOV3, IGROV1 and PA1) without *BRCA* mutation^46^ are intentionally selected to exclude defects of DNA damage repair. We reveal that the inflammatory response is significantly elevated in Olaparib‐resistant cells after long‐term PARPi treatment, which is likely attributed to the accumulation of DNA damage and activation of key inflammatory transcription factor NF‐κB. Surprisingly, A_2B_, among genes in inflammatory pathway and adenosine receptors, is remarkably increased in Olaparib‐resistant cells. Adenosine stimulation through A_2B_ forms a positive feedback loop to induce additional A_2B_ expression. Notably, PARPs are nicotinamide adenine dinucleotide (NAD)‐consumed enzymes^47^; PARP inhibition results in NAD accumulation.^48^ While NAD and ATP can be converted into adenosine,^49^ we presume that adenosine signalling may play a role in PARPi resistance. Coincidentally, A_2B_ is one of the adenosine receptors. All these findings suggest that the upregulated A_2B_ expression resulting from Olaparib‐induced inflammatory signalling confers the PARPi resistance in ovarian cancer cells.

Adenosine signalling through its four receptors has been reported to play an important role in cancer progression.^21^ Among these receptors, A_2B_ is widely expressed but has the highest activation threshold, which is hard to be activated under physiological conditions. However, under pathological conditions such as tumorigenesis, A_2B_ expression is upregulated to sense adenosine and activate adenosine signalling.^22^ Thereby, high A_2B_ expression in tumour patients predicts poorer prognosis.^33^, ^50^ Consistent with these findings, we also discover that the A_2B_ expression negatively correlates with the OS in ovarian cancer patients. The elevated expression of A_2B_ in Olaparib‐resistant cells (PEO1‐R, A2780‐R and OVCAR3‐R) promotes both cell growth and migration of ovarian cancer cells to exert its protumorigenic functions. Notably, although the pro‐tumour effects of A_2B_ signalling have been reported in certain types of cancer cells such as breast, bladder and head and neck cancers,^39^ its function in ovarian cancer cells, to our knowledge, is revealed for the first time. The enhanced protumorigenic role of A_2B_ contributes to the Olaparib resistance in ovarian cancer cells.

As a member of GPCRs, A_2B_ collaborates with G proteins to activate cAMP signalling. However, GPCR‐cAMP signalling is decreased in the resistant cells. Instead, the inflammatory signalling pathway is significantly enriched and the IL‐6‐STAT3 signalling is activated in Olaparib‐resistant cells. IL‐6 is a multifunctional cytokine that has been widely reported to promote tumorigenesis.^44^ Our results reveal that adenosine‐A_2B_ signalling in Olaparib‐resistant cells induces IL‐6 secretion and subsequent STAT3 activation, which promotes tumour cell growth. Given the important role of A_2B_‐IL‐6‐STAT3 signalling in Olaparib resistance, inhibition of this axis offers benefits for the anti‐tumour effects of Olaparib. Currently, a variety of STAT3 inhibitors have been developed and tested in clinical trials. For instance, C188‐9 is a potent STAT3 inhibitor and has been assessed in a clinical phase I study for advanced tumours (NCT03195699). In our study, C188‐9 can effectively suppress the growth of Olaparib‐resistant cells, whereas the combination of C188‐9 and Olaparib exhibits stronger anti‐tumour effects.

In conclusion, our work reveals an important role of A_2B_ in mediating Olaparib resistance in ovarian cancer cells by activating IL‐6‐STAT3 signalling pathway, highlighting a novel application of A_2B_ and STAT3 inhibition in defeating Olaparib resistance. With the advances of both A_2B_ inhibitors (TT‐4, TT‐702, GS‐6201, PBF‐1129, etc.) and STAT3 inhibitors (C188‐9, Napabucasin, GLG‐801, WP‐1066, PCUR‐101, etc.) in the clinic, combination of those drugs with PARP inhibitors may achieve better therapeutic outcomes in ovarian cancers. Future studies are also required to assess the dynamic changes of adenosine metabolism during Olaparib treatment and to explore other inflammatory pathways involved in Olaparib resistance.

## AUTHOR CONTRIBUTIONS

**Liqing Chi:** Data curation (equal); formal analysis (equal); investigation (lead); project administration (lead); writing – original draft (lead); writing – review and editing (lead). **Lin Huan:** Data curation (equal); formal analysis (equal); investigation (supporting); project administration (supporting); writing – original draft (supporting). **Chunyan Zhang:** Investigation (supporting); project administration (supporting); writing – review and editing (supporting). **Hanming Wang:** Investigation (supporting); resources (supporting). **Jian Lu:** Resources (lead); writing – review and editing (equal).

## CONFLICT OF INTEREST STATEMENT

The authors confirm that there are no conflicts of interest.

## Supporting information


FiguresS1–S3

## Data Availability

No data are available.
