# Supplementary material for: Adenosine receptor A2b confers ovarian cancer survival and PARP inhibitor resistance through IL‐6‐STAT3 signalling
Source: J Cell Mol Med. 2023 Jun 6;27(15):2150–64. doi: 10.1111/jcmm.17802 (PMC10399543; doi:10.1111/jcmm.17802)
Supplement: Supplementary file 1 — FiguresS1–S3 [file JCMM-27-2150-s001.docx]

**Figure S1. Increased inflammatory signalling pathway and A_2B_ expression in Olaparib-resistant ovarian cancer cells.**

**
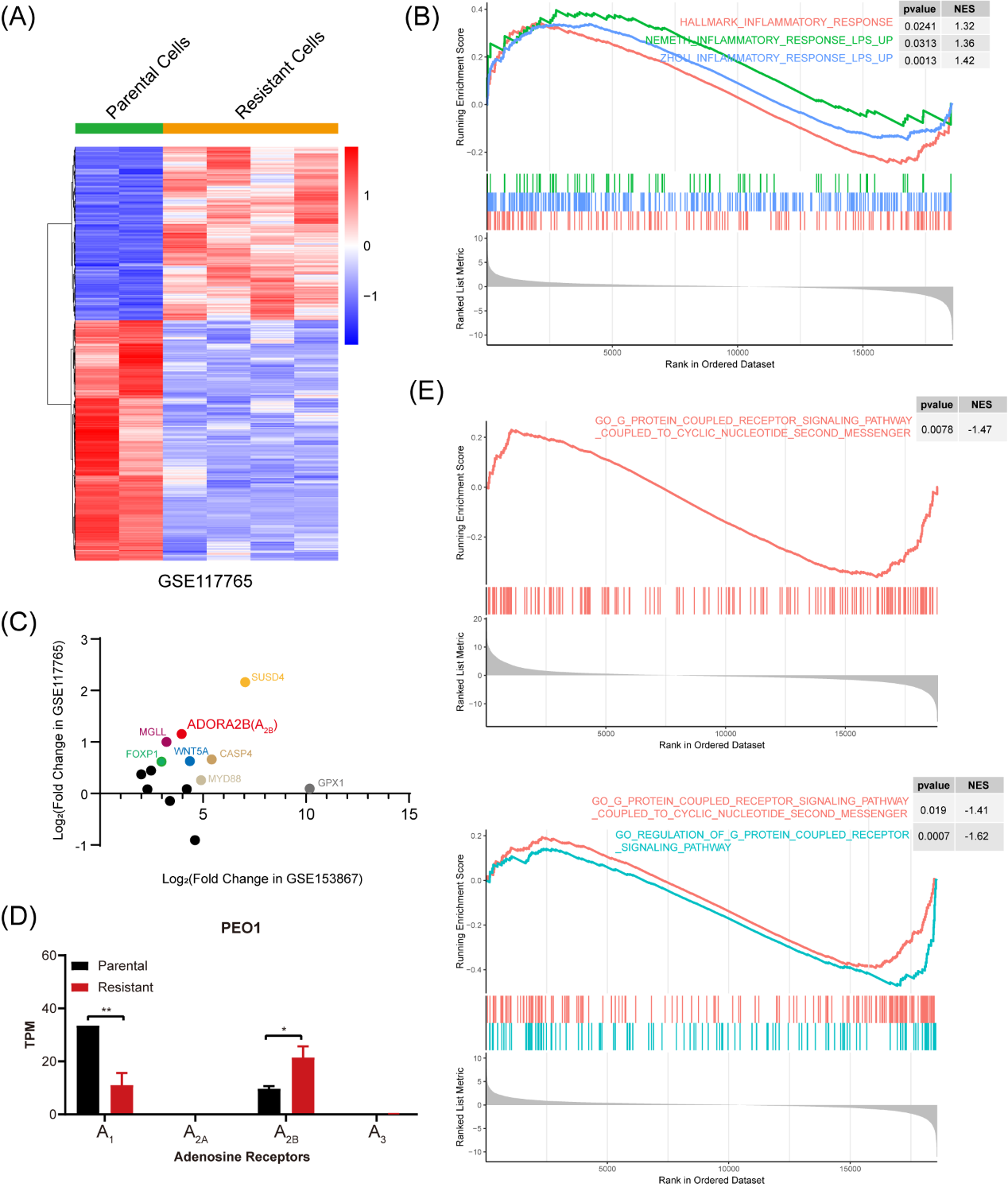
**

**Figure S2. Establishment of Olaparib-resistant ovarian cancer cell line OVCAR3-R.**

**
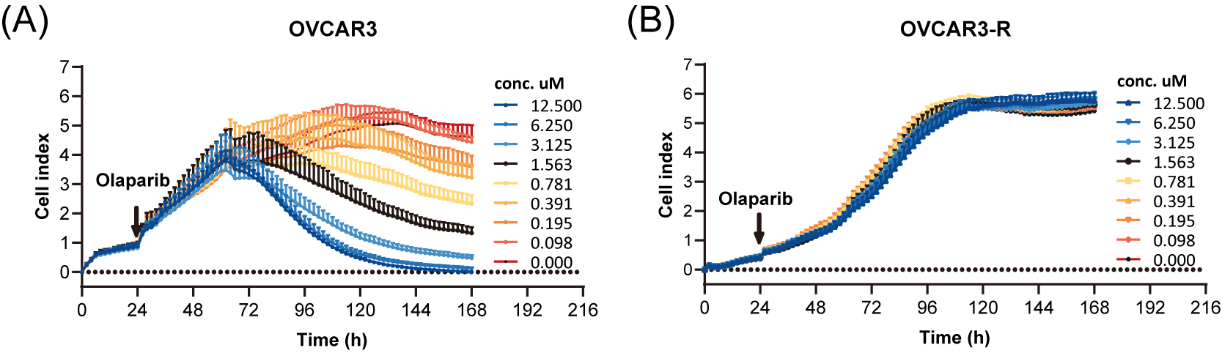
**

**Figure S3. A_2B_ signalling promotes ovarian cancer cell growth.**

**
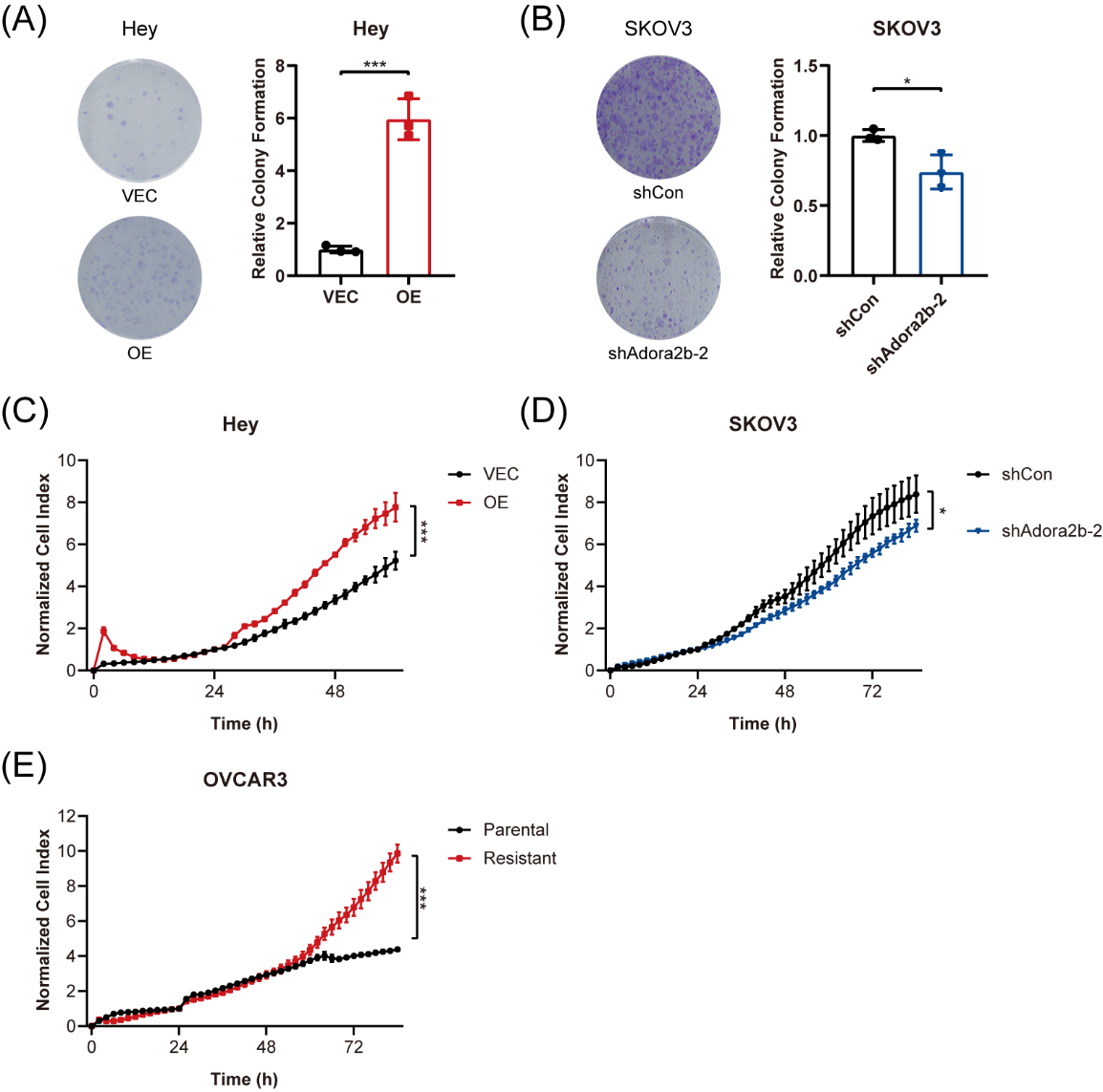
**

**Figure legends**

**Figure S1. Increased inflammatory signalling pathway and A_2B_ expression in Olaparib-resistant ovarian cancer cells.** (A) Heatmap illustrating differentially expressed genes between parental (*n=*2) and Olaparib-resistant (*n=*4) PEO-1 cells. *p* value was calculated using Wilcoxon test, genes with |Log_2_ (Fold Change)|>1 and p < 0.05 were chosen for heatmap. (B) Gene set enrichment analysis (GSEA) of inflammatory response pathway using GES117765 (PEO1 cell) data. (C) Plot showing changes of genes in Gene Ontology geneset, and p value was calculated using Wilcoxon test. (D) TPM values of ADO receptor genes in parental and Olaparib-resistant PEO1 cells were shown. (E) GSEA of GPCR signalling pathway using GSE153867 (top) and GES117765 (bottom) data. Data are representative of three independent experiments shown as the mean ± SD. Statistical testing is depicted as two-tailed unpaired Student’s *t* test. **p*<0.05, ***p*<0.01, ****p*<0.001.

**Figure S2. Establishment of Olaparib-resistant ovarian cancer cell line OVCAR3-R.** Cell growth of parental OVCAR3 (A) and Olaparib-resistant OVCAR3-R cells (B) treated with indicated doses of Olaparib at different time was detected using a real-time cell analyser (*n=*4).

**Figure S3. A_2B_ signalling promotes ovarian cancer cell growth.** (A) Cell growth of control (VEC) and A_2B_-overexpressed (OE) Hey cells was detected by colony formation assay (*n=*3). The representative images were on the left, and the statistical graph was on the right with normalization to VEC group. (B) Cell growth of SKOV3 cells with (shAdora2b-2) and without (shCon) A_2B_-knockdown was detected by colony formation assay (*n=*3). The representative images were on the left, and the statistical graph was on the right with normalization to shCon group. (C) Cell growth of control (VEC) and A_2B_-overexpressed (OE) Hey cells was detected using a real-time cell analyser (*n=*3). The cell index was normalized to that at 24 hours after cells seeded onto plates, and *p* value was calculated using two-way ANOVA. (D) Cell growth of SKOV3 cells with (shAdora2b-2) and without (shCon) A_2B_-knockdown was detected using a real-time cell analyser (*n=*3). The cell index was normalized to that at 24 hours after cells seeded onto plates, and *p* value was calculated using two-way ANOVA. (E) Cell growth of parental (OVCAR3) and Olaparib-resistant (OVCAR3-R) cells was detected using a real-time cell analyser (*n=*3). Data are representative of three independent experiments shown as the mean ± SD. Statistical testing is depicted as two-tailed unpaired Student’s *t* test. **p*<0.05, ***p*<0.01, ****p*<0.001.
